# Establishment of TP53-knockout canine cells using optimized CRIPSR/Cas9 vector system for canine cancer research

**DOI:** 10.1186/s12896-018-0491-5

**Published:** 2019-01-03

**Authors:** Kiyoung Eun, Min Gi Park, Yeon Woo Jeong, Yeon Ik Jeong, Sang-Hwan Hyun, Woo Suk Hwang, Sung-Hak Kim, Hyunggee Kim

**Affiliations:** 1 Department of Biotechnology, College of Life Sciences and Biotechnology, Korea University, 145 Anam-ro, Seongbuk-gu, Seoul, 02841 Republic of Korea; 2 Sooam Biotech Research Foundation, 64 Kyungin-ro, Guro-gu, Seoul, 08359 Republic of Korea; 3 Laboratory of Veterinary Embryology and Biotechnology, College of Veterinary Medicine, Chungbuk National University, Cheongju, 28644 Republic of Korea; 4 Department of Animal Science, College of Agriculture and Life Sciences, Chonnam National University, Gwangju, 61186 Republic of Korea; 5 Gwangju Center, Korea Basic Science Institute, Gwangju, 61186 Republic of Korea

**Keywords:** CRISPR/Cas9, Dog, TP53, Knockout, Cancer

## Abstract

**Background:**

Genetic engineering technology such as clustered regularly interspaced short palindromic repeats (CRISPR)/Cas9 system provides a powerful tool for developing disease models and determining gene functions. Recent interests in canine cancer models have highlighted the necessity of developing genetic engineering tools for dogs. In this study, we attempted to generate optimized CRISPR/Cas9 system to target canine tumor protein 53 (*TP53*), one of the most crucial tumor suppressor genes, to establish TP53 knockout canine cells for canine cancer research.

**Results:**

We constructed CRISPR/Cas9 vectors using each of three *TP53* gene-targeting guide RNAs (gRNAs) with minimal off-target potential. After transfection, we obtained several clones of *TP53* knockout cells containing “indel” mutations in the targeted locus which had infinite cellular life span, resistance to genotoxicity, and unstable genomic status in contrast to normal cells. Of the established TP53 knockout cells, TP53KO#30 cells targeted by TP53 gRNA #30 showed non-cancerous phenotypes without oncogenic activation both in vitro and in vivo. More importantly, no off-target alteration was detected in TP53KO#30 cells. We also tested the developmental capacity of TP53 knockout cells after application of the somatic cell nuclear transfer technique.

**Conclusions:**

Our results indicated that *TP53* in canine cells was effectively and specifically targeted by our CRISPR/Cas9 system. Thus, we suggest our CRISPR/Cas9-derived canine *TP53* knockout cells as a useful platform to reveal novel oncogenic functions and effects of developing anti-cancer therapeutics.

**Electronic supplementary material:**

The online version of this article (10.1186/s12896-018-0491-5) contains supplementary material, which is available to authorized users.

## Introduction

Canine cancer is among the most common causes of death of pet dogs. According to a report from the Swiss Canine Cancer Registry, 51.83% (67,943 of 126,693) of dogs were diagnosed with various cancers from 1955 to 2008 [1]. Although canine cancer therapy has considerably improved and multiple genes were identified as candidate biomarkers to detect and cure the cancer, the prognosis of various canine cancer patients remains poor [2]. Understanding molecular aspects of canine oncology will improve clinical outcomes. Canine cell models susceptible to tumorigenesis are the most essential materials to elucidate the molecular backgrounds underlying carcinogenesis and to find novel therapeutic targets. Moreover, recent interests in canine cancer models for use in the translational research field have also highlighted the necessity of developing research tools for dogs [3].

Tumor protein 53 (*TP53*) gene is a key regulator in cell cycle arrest, cellular senescence, and apoptotic responses induced by various stresses [4]. *TP53* is also known as the most crucial tumor suppressor gene and its mutation frequency was over one-third of pan-cancer patients [5, 6]. So, its importance in cancer initiation and progression, and in therapeutics has been well identified by numerous studies [7]. Like in human cancer, genetic alteration in *TP53* gene was frequently observed in various canine cancer including lymphoma and mammary cancer [8, 9]. So, canine *TP53* modulating tools and canine experimental model of TP53 deficiency are the most fundamental requirement to study canine cancers.

Recently, the type II clustered regularly interspaced short palindromic repeats (CRISPR)/Cas9 system, an RNA-guided nuclease-mediated adaptive immune system of *Streptococcus pyogenes* against phages and viruses, was reconstituted in eukaryotic cells via codon optimization and the unification of two CRISPR RNA components, the guide RNA (gRNA) and trans-activating CRISPR RNA, into a single guide RNA [10–13]. Double strand breaks (DSBs) generated by its two nuclease domains, HNH and RuvC, are then restored via one of two cellular repair systems: non-homologous end-joining and homology-directed repair pathways. The former produces a random insertion or deletion (indel) mutation around the DSB site, while the latter introduces precise insertion of an intended DNA sequence from a designed donor template [14]. However, the potential off-target activity of the RNA-guided CRISPR/Cas9 system causing unintended genetic alterations is a major concern in basic and clinical applications. [15]. Therefore, minimizing the off-target potential of this system is critical for obtaining precise results.

In this study, we constructed a CRISPR/Cas9 vector system for canine *TP53* with minimum off-target potential and *TP53* knockout canine fibroblasts using the system, and finally evaluate their utilities in cancer studies.

## Results

### Construction of CRISPR/Cas9 systems for canine TP53 gene knockout

**Fig. 1:**
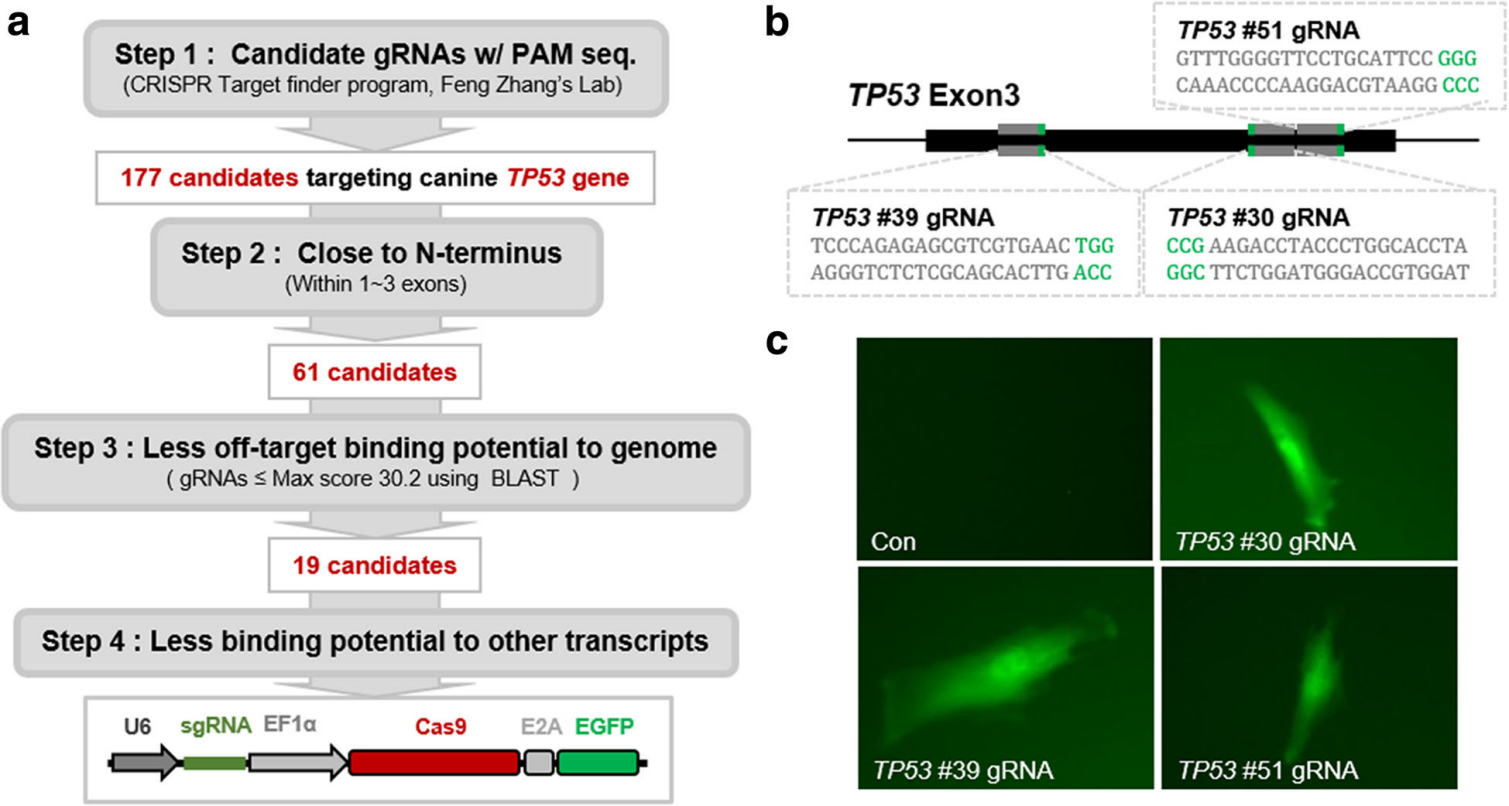
Construction of CRISPR/Cas9-mediated canine *TP53* gene knockout (KO) system. **a** Procedure to design and select guide RNAs (gRNAs) specifically targeting canine *TP53*. Step 1: Candidate gRNAs for all exons of the gene were searched using the CRISPR Target Finder Program. Step 2: Candidates for 1–3 exons were selected to avoid partial functional protein production. Steps 3 and 4: gRNAs with less binding potential to the canine genome and transcripts were sorted according to the BLAST alignment algorithm. The final three gRNAs of *TP53* were inserted into the CRISPR/Cas9 expression vector. **b** Sequences of the three gRNAs targeting canine *TP53* exon 3. **c** Transient transfection and Cas9 gene expression was detected based on enhanced green fluorescence proteins (EGFP) reporter transgene, which was cleaved from the Cas9 protein via 2A peptide-mediated self-cleavage

**Fig. 2:**
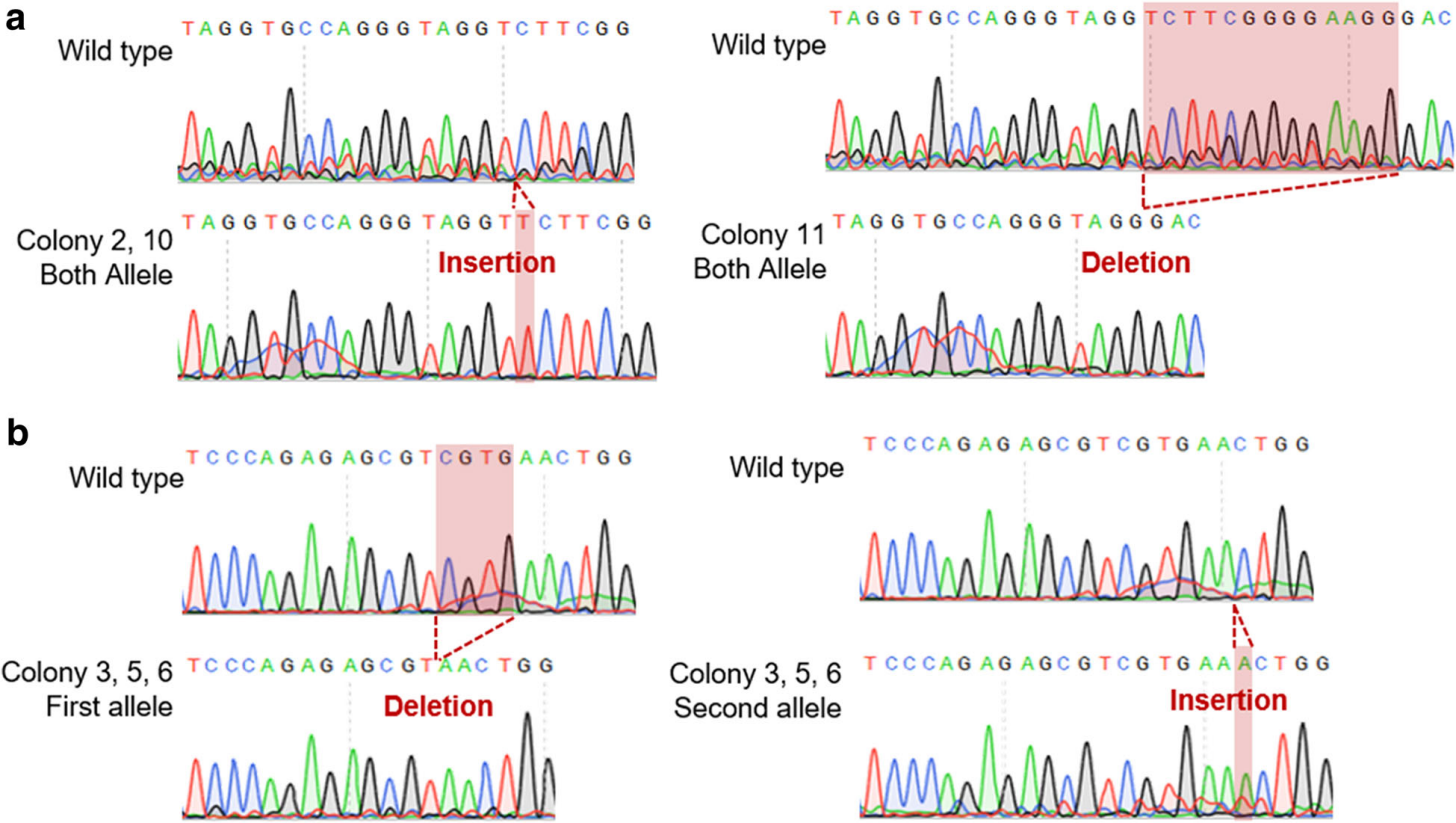
Sequencing analysis of CRISPR/Cas9-mediated *TP53* knockout (KO) canine fatal fibroblasts. **a** Nucleotide sequences of targeted *TP53* genomic loci of cells treated with *TP53* gRNA #30 (colonies #2, #10, and #11) and **b** *TP53* gRNA #39 (colonies #3, #5, and #6)

To target the canine *TP53* locus via the CRISPR/Cas9 system, we selected three gRNAs with the lowest off-target potentials (Fig. 1a, b). These gRNAs were applied to our CRISPR/Cas9 expression vector and transiently transfected into canine fetal fibroblast cells (K9 Fetus 1), in which cellular senescence phenotypes appeared at passages 6–8 (Fig. 1c). A previous study suggested that knockout (KO) of *TP53* extends the limited cellular life span of mammalian somatic cells [4]. Thus, after culturing the control cells until they were senescent, consecutively proliferating cell colonies were obtained from cells targeted by *TP53* gRNA #30 and #39 (Additional file 1: Figure S1). Next, sequencing of each target locus was performed using morphologically healthy colonies (#2, #10, #11 from gRNA *TP53* #30; and #3, #5, #6 from *TP53* #39). Cells from gRNA #51 were excluded because of their abnormal morphology and relatively low growth rate (Additional file 1: Figure S1). All analyzed cells contained an insertion or deletion mutation causing a frame shift at the targeted locus in colonies from cells of *TP53* gRNA #30 and *TP53* gRNA #39 (Fig. 2a, b). Colonies with the same mutation may have originated from the same parental cell during plate transfer. Therefore, these data showed that two of the three constructed canine *TP53* targeting CRISPR/Cas9 systems effectively knocked out *TP53*.

### TP53 KO canine fibroblasts were immortalized and acquired resistance against genotoxic stress

**Fig. 3:**
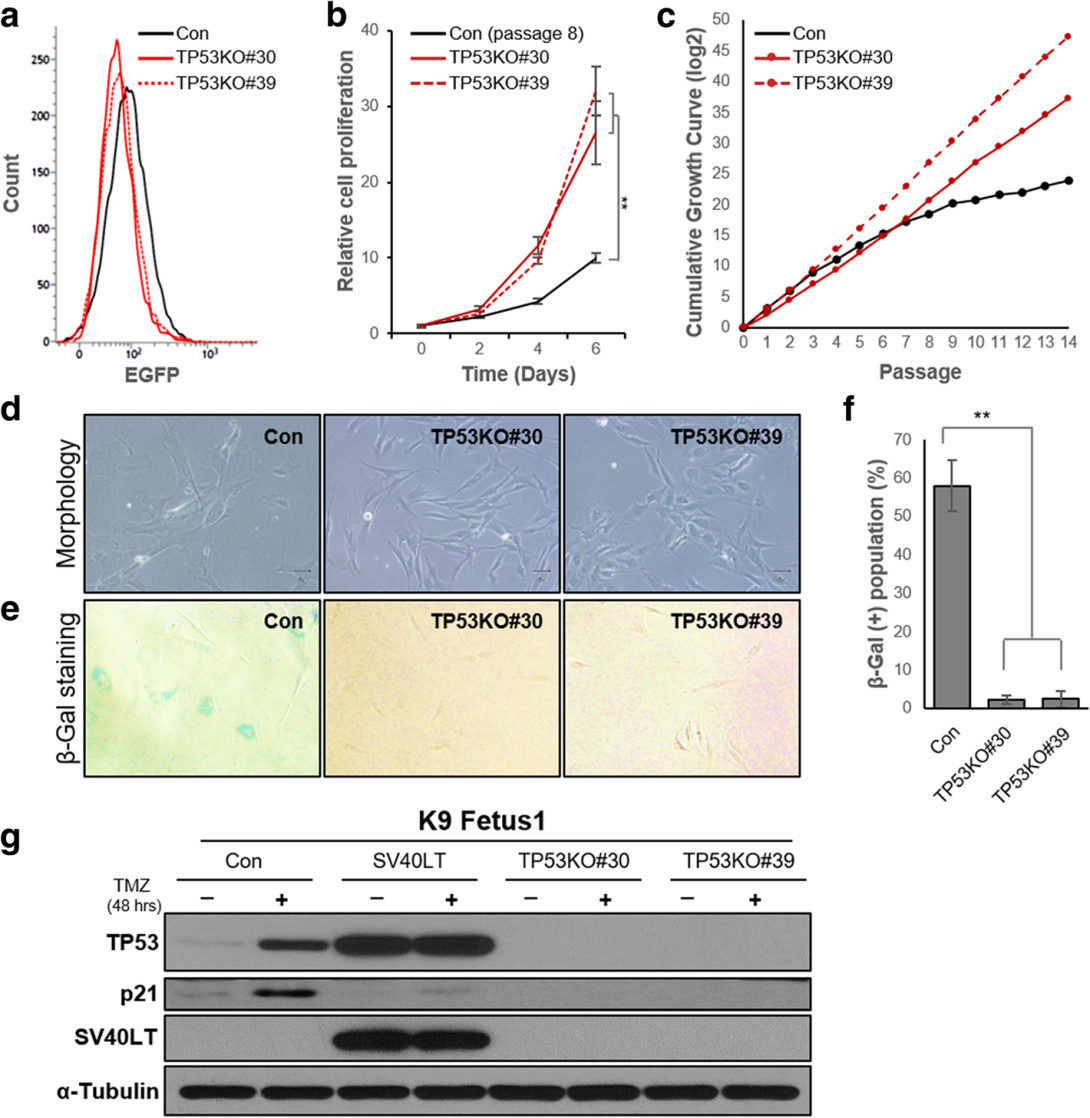
Cellular characteristics of canine fibroblasts immortalized by CRISPR/Cas9-mediated *TP53* knockout (KO). **a** No enhanced green fluorescent protein (EGFP) expression was detected by fluorescence activated cell sorting (FACS) analysis in both TP53KO#30 and TP53KO#39 cell lines. **b** Relative proliferation rates of each cell lines (× 15,000 cells). **c** Cumulative growth curve data of the control (early passage 2) and two *TP53* KO cells were obtained after additional 14 consecutive passages in culture. **d** Representative images showing the cellular morphologies of the control and two *TP53* KO cells at the end time-point (passage 14) of cumulative growth counting analysis. **e** Representative images of the control and two *TP53* KO cells stained with senescence associated β-galactosidase (SA-β-gal) at the end time-point of cumulative growth counting analysis. **f** Percentage of SA-β-gal-positive cells in each group. **g** Immunoblot data showing TP53, p21, and SV40LT protein levels in the control, SV40LT-transduced, TP53KO#30, and TP53KO#39 cells grown in the absence or presence of 100 μM TMZ for 48 h. α-Tubulin was used as the loading control. **, *p* < 0.01

To identify the characteristics of *TP53* KO canine fibroblasts, we selected cell lines of colony #10 from gRNA#30 and #6 from gRNA#39, which were named as TP53KO#30 and TP53KO#39, respectively. They did not express EGFP, indicating non-integration of the CRISPR/Cas9 vector into the host genome (Fig. 3a). Initially, their growth rates were measured every 3 days for up to an additional 15 passages. The growth rates of TP53KO#30 and TP53KO#39 cell lines were faster than the late passage (p8) control cells (Fig. 3b). Cumulative growth curve data showed that their proliferative capacities were maintained (Fig. 3c). Unlike normal fibroblasts entering senescence, the cells showed non-senescent phenotypes even after late passages. However, only the TP53KO#30 cell line maintained its initial morphology, while TP53KO#39 did not (Fig. 3d). To identify senescent populations, senescence-associated β-galactosidase (SA-β-gal) staining was conducted. An SA-β-gal-positive population was rarely detected, even at late passages (Fig. 3e, f). Although the morphology of TP53#39 cells changed during long-term in vitro culture, these data show that both of canine *TP53* KO fibroblasts had become immortal.

The TP53 protein expressed by *TP53* is engaged in cell cycle arrest and DNA repair mechanisms after DSBs; therefore, its deficiency is closely related to resistance to ionizing radiation or genotoxic chemicals [16, 17]. During this process, the cyclin-dependent kinase inhibitor p21 is transcriptionally upregulated by activated TP53 protein and functions in DNA damage-induced cell arrest [18]. Thus, we treated the TP53KO#30 and TP53KO#39 cell lines with temozolomide (TMZ), a DNA alkylating agent, to examine whether *TP53* function was intact. As determined by the levels of p21 protein in each cell after TMZ treatment for 48 h, in sharp contrast to K9 fetus 1 control cells, p21 protein was not apparently induced by TMZ in *TP53* KO cells and cells in which *TP53* was inactivated by SV40LT (Fig. 3g). These results indicate that both CRISPR/Cas9-mediated *TP53* KO fibroblast lines are resistant to DNA damage, which is a typical characteristic of *TP53*-deficient cells.

### Tumorigenic potential testing of CRISPR/Cas9-mediated TP53 KO canine cells

**Fig. 4:**
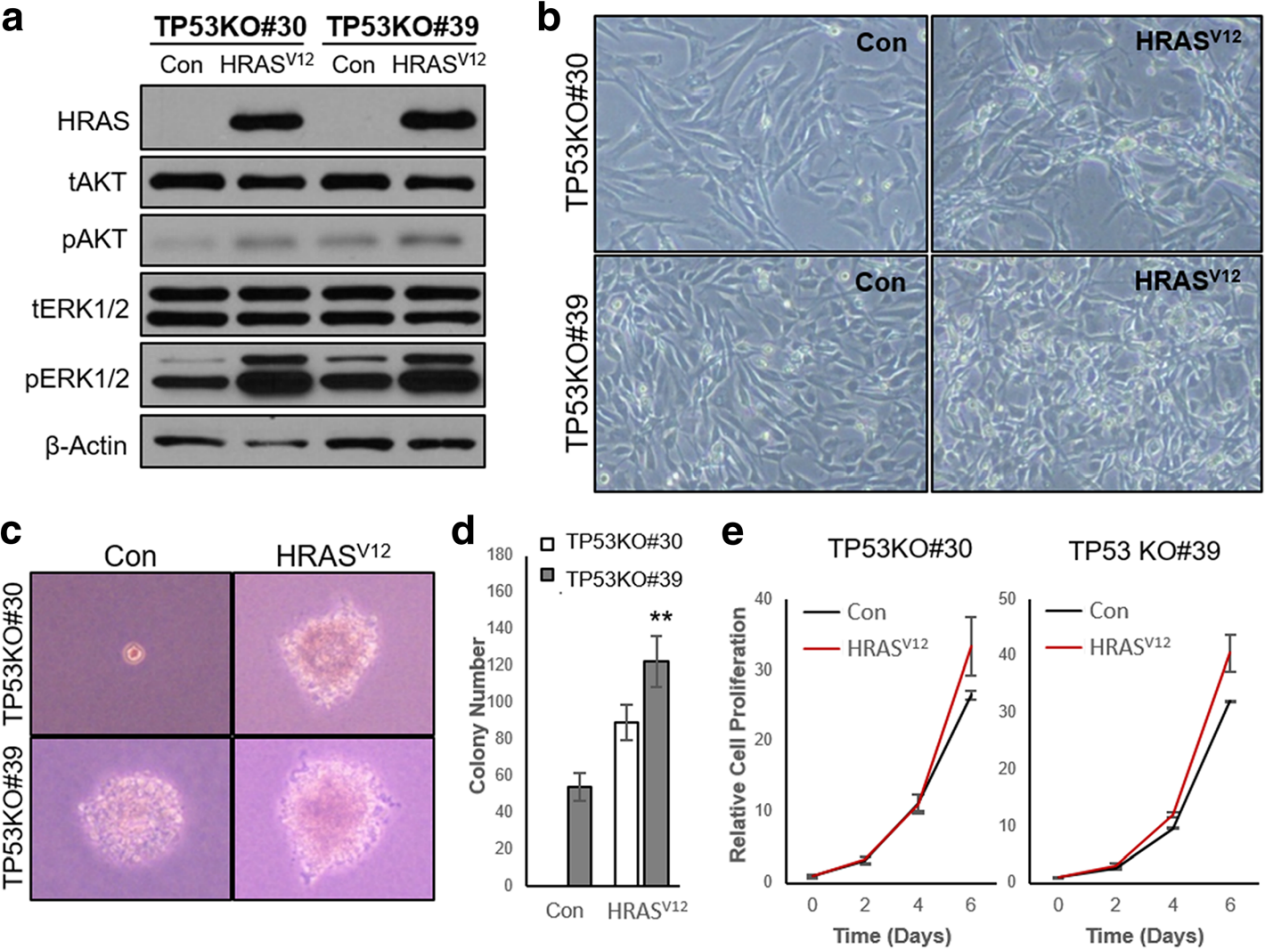
Non-tumorigenic potential of CRISPR/Cas9-mediated Tp53KO#30 canine fibroblasts in vitro. **a** Immunoblot data showing HRAS, total AKT (tAKT), phosphorylated AKT (pAKT), tERK1/2, and pERK1/2 protein levels in TP53KO#30, TP53KO#39, and their corresponding HRAS^V12^-transduced cells. β-Actin was used as the loading control. **b** Representative images showing the cellular morphologies of TP53KO#30, TP53KO#39, and their corresponding HRAS^V12^-transduced cells. **c** Representative images showing the generated colonies of TP53KO#30, TP53KO#39, and their corresponding HRAS^V12^-transduced cells grown in semi-solid agar cultures for 32 days. **d** Quantitative data of colony numbers in semi-solid agar cultures. **e** Proliferation rates of TP53KO#30, TP53KO#39, and their corresponding HRAS^V12^-transduced cells

**Fig. 5:**
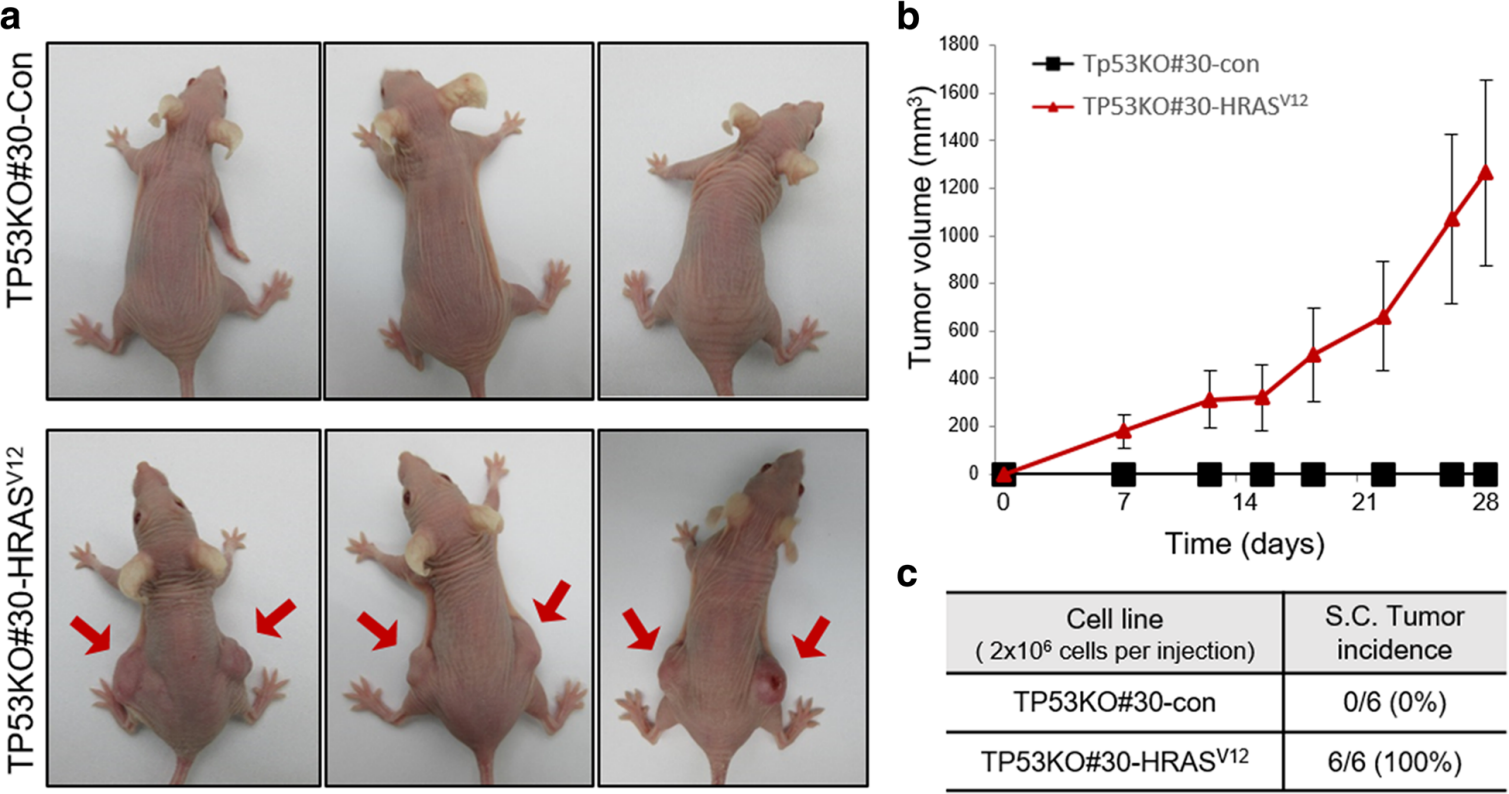
In vivo subcutaneous tumorigenic assay. **a** Images of TP53KO#30-Con-injected mice and TP53KO#30-HRAS^V12^-injected mice at day 28. Red arrows indicates developed subcutaneous tumors. **b** Tumor growth rates of TP53KO#30 and TP53KO#30-HRAS^V12^ cells. **c** Tumor incidence of indicated cells. *, *P* < 0.05; **, *P* < 0.01

**Fig. 6:**
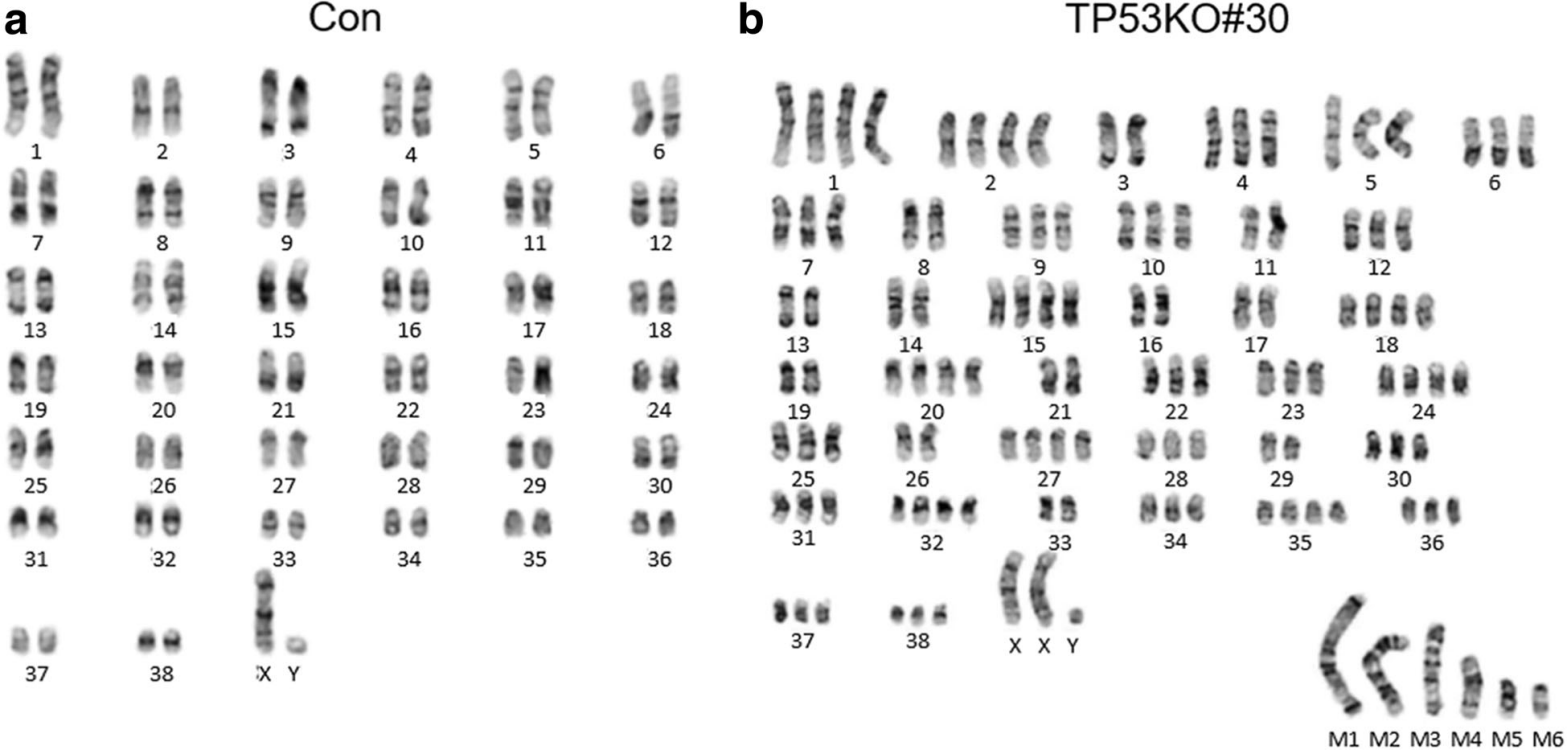
Karyotype analysis. **a** Karyotypes of normal canine fibroblasts and **b** of TP53KO#30 cells. M indicates marker chromosome

Next, we examined whether our CRISPR/Cas9-mediated *TP53* KO canine cells acquired tumorigenic potential. Although alterations in *TP53* are a crucial genetic events in various cancers, these events are not sufficient to induce cellular transformation [19]. Along with deregulation of tumor suppressors, multiple genetic events including activation of oncogenic signaling are required for cancer development [20, 21]. Based on this, HRAS^V12^, an oncogenic mutant of human HRAS, was used to compare the cancerous properties of the cells. We found that HRAS^V12^ activated both the mitogen-activated protein kinase and PI3K-AKT pathways in canine cells, as determined by increases in phosphorylated AKT (pAKT) and ERK1/2 (pERK1/2) (Fig. 4a). Importantly, TP53KO#30 was morphologically normal, whereas TP53KO#39 proliferated in multilayers on culture plates, similar to HRAS^V12^ overexpressing cells (Fig. 4b). Additionally, only the TP53KO#30 cell line did not grow anchorage-independently in semi-solid agar culture (Fig. 4c, d), with no apparent difference in proliferation rates compared to TP53KO#30-HRAS^V12^ cells (Fig. 4e). Thus, the TP53KO#39 cell line was transformed, as these properties are associated with tumorigenicity [22]. The transformation of TP53KO#39 cells may be attributable to an off-target effect of the CRISPR/Cas9 system [23]. Therefore, we selected several genes with high off-target potential based on their correspondence with the two *TP53* KO gRNA sequences (Additional file: Table S1) and detected whether mutations had been generated using a surveyor assay. The results showed that TP53KO#39 had a mutation in *TP53* and in *TFE3* (Additional file 1: Figure S2). These data indicate that TP53KO#39 cells were affected by the non-specific endonuclease activity of *TP53* gRNA #39-loaded Cas9. Moreover, TP53KO#30 cells showed no in vivo tumorigenic potential without oncogenic activation (Fig. 5a-c). Karyotype analysis revealed that TP53KO#30 cells had an aberrant chromosome status, which may have been caused by disruption of *TP53*-governing genome protective mechanisms (Fig. 6a, b). These data suggest that TP53KO#30 cells, but not TP53KO#39 cells, did not acquire tumorigenic potential despite their aneuploidy.

### Developmental capacity of TP53KO#30 cells after somatic cell nuclear transfer (SCNT)

Recently, cloned dogs were successfully produced via the somatic cell nuclear transfer (SCNT) technique [24]. A previous study showed that SCNT embryos derived from *TP53*^−/−^ breast cancer cells, but not malignant primary cancer cells, successfully developed into blastocysts [25]. We predicted that examining the developmental capacity of somatic cells after SCNT would provide novel criteria for cellular disposition. Thus, we attempted to generate TP53KO dogs using our TP53KO#30 cells by SCNT. In total, 500 nuclear transferred embryos (218 from the first trial and 282 from the second trial, with fusion rates of 50.5 and 42.3%, respectively) were fused successfully with enucleated oocytes. These embryos were transferred into 32 surrogate dogs. Unexpectedly, all SCNT embryos failed to generate a successful pregnancy after embryo transfer (Table 1).

**Table 1:** Pregnancy outputs from cloned embryos according to cell line

Cell type	Nuclear transfer				Embryo transfer		
	No. of oocyte donors dogs	No. of			No. of surrogate dogs	No. of pregnancies (%)^a^	
		Oocyte subjected to NT	Oocyte fused & transferred (%)^c^	Offspring born (%)^d^		On Day 30	Full Term^b^
K9 Fetus 1 (control)	14	121	109 (90.1)	3 (2.8)^e^	7	3 (21.1)^e^	3 (21.1)^e^
Tp53KO#30 (1st trial)	43	491	218 (50.5)	0 (0.0)	15	0 (0.0)	0 (0.0)
Tp53KO#30 (2nd trial)	66	770	282 (42.3)	0 (0.0)	17	0 (0.0)	0 (0.0)

^a^ Pregnancy was detected using real-time ultrasonography. Pregnancy rate was calculated based on the number of pregnancies observed out of the total number of surrogates that were transferred with reconstructed embryos (fused oocytes)

^b^ Full term pregnancy refer to pregnancy that successfully produced live offspring

^c^ Fusion rate was calculated based on the number of fused oocytes out of the total oocytes that were subjected to nuclear transfer (NT)

^d^ The offspring born were calculated based on the total number of reconstructed embryo transferred

^e^ Superscripts in different rows in the same column differ significantly (*P* < 0.05)

## Discussion

Despite advances in veterinary science, cancer remains a leading cause of death of pet dogs. Combining clinical studies and appropriate mechanistic studies of canine cancers will promote the development of advanced drugs and improve clinical outcomes. Recently, spontaneous canine cancer models have attracted the attention of the translational research field because of the various similarities in oncogenetic characteristics, histological features, and response to conventional therapies in humans [3]. However, few research materials such as canine cell lines, genetic engineering tools, antibodies, and even bioinformatics database for canine models. Thus, various experimental tools for canine cancer research are urgently needed.

In this study, we designed CRISPR/Cas9 vector systems for canine *TP53*, an important tumor suppressor gene. CRISPR/Cas9 of *TP53* gRNA #30 and #39, but not #51, effectively targeted canine *TP53*. We also examined the proliferation capacities, morphological changes, and cellular transformation of canine fetal fibroblast cells targeted by *TP53* gRNA #30 and #39. In contrast to TP53KO#30 cells, TP53KO#39 cells showed abnormal phenotypes. As observed in an early *TP53*-knockout mouse model, depletion of *TP53* alone is not sufficient to induce transformation into cancerous cells [26]. Thus, abnormal characteristics of TP53KO#39 cells may be caused by undesired genetic alterations generated from off-target effects of the CRISPR/Cas9 or attenuation of genetic stability governed by *TP53*. One possibility is that a mutation in *TFE3*, a possible off-target gene for *TP53* gRNA #39, caused cellular transformation. Therefore, our results suggest that the sequence of *TP53* gRNA #30, which has a lower potential for off-target effects, is an effective tool for targeting canine *TP53*, and TP53KO#30 cells are *TP53*-targeted canine cells without off-target gene alterations. This first successful application of the CRISPR/Cas9 system in canine models may promote the development of various genetic engineering tools for a range of purposes in canine models.

*TP53* deficiency is related to high incidences of human malignancies including lymphomas, soft-tissue carcinomas, or even Li-Fraumeni syndrome; therefore, *TP53* KO mice have been used as a genetic background model for specific cancers, including glioma, ovarian cancer, medulloblastoma, and lung cancer by modulating genes of interest such as KRAS, NF1, or MYC [27–30]. These cancer models have provided critical information of regarding oncogenic signaling during tumor initiation and progression. Additionally, many reports have utilized cancer cell models, generated by modulating *TP53* and/or oncogenes, to evaluate the efficacy of developing drug [31, 32]. Genetic engineering tools targeting *TP53* and *TP53*-deficient cell lines are among the most fundamental experiment materials useful for various cancer studies, particularly for studying the pathological functions of unidentified oncogenes in canine cancers and developing therapeutics.

*TP53* KO alone does not cause any critical problems in early mouse development [26]; therefore, if TP53KO#30 cells exhibit normal characteristics, it should be possible to generate *TP53* KO dogs via SCNT. Thus, we attempted to generate *TP53* KO dogs via SCNT using TP53KO#30 cells as nuclear donor cells. However, the SCNT embryos derived from TP53KO#30 cells failed to initiate pregnancy in surrogate dogs. These failures in embryo development and pregnancy may have been caused by chromosome abnormalities in TP53KO#30 cells [33, 34]. Chromosomal missegregation did not occur frequently during early embryogenesis of *TP53* KO mice; however, a tetraploid cell population was increased in postnatal *TP53* KO mice [35]. *TP53* loss in normal human fibroblasts and even epithelial cancer cells does not always result in aneuploidy [36]. These previous studies showed that disruption of *TP53-*governing genetic defense mechanisms leads to chromosomal abnormality or genetic alterations by chance during constant proliferation. Thus, immediate SCNT after transfection of our CRISPR/Cas9 system may be useful for producing *TP53* KO dogs to avoid the occurrence of chromosomal abnormalities. Additionally, it may be more scientifically valuable to produce transgenic canine models of specific cancers by combining our canine *TP53* targeting system with tissue or cell type-specific expression systems. Therefore, our *TP53*-targeting system may be utilized to produce *TP53*-modulated canine transgenic models in further studies.

## Conclusions

We constructed an optimal CRISPR/Cas9-mediated *TP53* targeting system for dogs with minimal off-target potential and established *TP53* KO canine cells, TP53KO#30, by applying our system. Because of the weak off-target activity, the established TP53KO#30 cells showed typical characteristics of *TP53*-deficient cells without any cancerous properties. Therefore, our CRISPR/Cas9-mediated *TP53* targeting system and TP53KO#30 cells can be utilized as a useful experimental materials for producing in vitro and in vivo canine cancer models to study the biology of canine.

## Methods

### Cell culture

*Canis lupus familiaris* canine fetal fibroblast cells (K9 Fetus 1), were maintained in Dulbecco’s modified Eagle’s medium (DMEM; Life Technologies, Carlsbad, CA, USA) supplemented with 10% fetal bovine serum (FBS; Life Technologies), 1% Glutamax (Life Technologies), 1% minimum essential medium non-essential amino acids (Life Technologies), 1% antibiotic-antimycotic (Life Technologies), and 0.1% 2-mercaptoethanol (Life Technologies).

For the temozolomide (TMZ; Sigma Aldrich, St. Louis, MO, USA) treatment experiment, cells were harvested using Trypsin-EDTA, counted, and reseeded at a density of 1 × 10^6^ cells per 100-mm culture plate. Twelve hours later, 100 nM of TMZ was added to each well.

### Guide RNA design

To design gRNAs targeting the canine *TP53* locus, all candidates with NGG-protospacer adjacent motif (PAM) sequences in all exons of the gene were identified using Feng Zhang laboratory’s Target finder program (https://zlab.bio/guide-design-resources). The program’s automatic off-target screening system was not available for dog species; therefore, we narrowed the results using the following steps. First, gRNAs near the N-terminus within the third exon of each gene were selected because their partial functional products could be produced if their functional domains were involved. Next, candidates with a lower binding potential compared to other genomic regions (gRNAs ≤ max score 30.2), particularly to coding sequences, were identified using the Basic Local Alignment Search Tool (BLAST) to avoid off-target alterations. Nineteen gRNAs for *TP53* were selected using this process and the final three gRNAs were selected based on their lowest non-specific binding potential, in accordance with the Max score determined using the BLAST algorithm.

### Vector construction

To construct a CRISPR/Cas9 plasmid vector targeting canine *TP53*, the U6-stuffer-hSpCas9 sequence was digested with *Kpn*1 (blunted by *Klenow* fragment) and *Bam*H1 from lentiCRISPRv2 (#52961; Addgene, Cambridge, MA, USA) [37]. The fragment was inserted into the *Nru*1 and *Bam*H1 sites of pcDNA3.1(+) (Thermo Fisher Scientific, Waltham, MA, USA). The enhanced green fluorescence protein (EGFP) sequence was digested with *EcoR1* and *Not1* from pEGFP-N2 (Clontech Laboratories Inc., Mountain View, CA, USA) and the synthesized E2A peptide sequence was inserted into the pcDNA3.1-CRISPR plasmid. Finally, the three designed gRNA sequences were synthesized and inserted into the two *Bsm*B1 sites flanking the stuffer sequence.

### Construction of canine TP53 KO fibroblasts

CRISPR/Cas9 vectors targeting *TP53* were transfected transiently using Polyexpress**™** (Excellgene, Rockville, MD, USA) into K9 fetus 1 cells at passage 1 according to the manufacturer’s instructions. The cells were then cultured and transferred into culture plates until untransfected cells showed senescence phenotypes. Finally, cell colonies with a normal cellular morphology and extended life span were separated and maintained independently.

### Semi-solid agar culture assay

A 1.5 mL lower layer of 0.72% agar in DMEM containing 10% FBS was added to each well of a 6-well plate and allowed to solidify at room temperature (19–21 °C). Cells tested by the semi-solid agar assay were suspended in a plating layer (1.5 mL) of 0.28% agar in DMEM containing 10% FBS and added to the wells. The agar was allowed to solidify at room temperature and then incubated at 37 °C in a humidified CO_2_ incubator. To prevent the agar from drying, 500 μL of DMEM containing 10% FBS was added to each well every 2–3 days.

### Cell growth counting

For cell proliferation analysis, 1.5 × 10^4^ K9 fetus 1 control cells and *TP53* KO cells were seeded into 6-well plates and cultured in the presence of 10% FBS for 6 days. Cells were harvested via trypsinization at various times and stained with Trypan blue. Viable cells were counted using a hematocytometer and inverted microscope. The number of cells was averaged over three independent experiments.

### Western blotting

For western blot analysis, whole cell extracts were prepared in radioimmunoprecipitation assay lysis buffer (150 mM NaCl, 1% NP-40, 0.1% SDS, and 50 mM Tris, pH 7.4) containing 1 mM β-glycerophosphate, 2.5 mM sodium pyrophosphate, 1 mM NaF, 1 mM Na_3_VO_4_, and a protease inhibitor cocktail (Roche, Basel, Switzerland). Protein levels were quantified using the Bradford assay reagent (Bio-Rad, Hercules, CA, USA) according to the manufacturer’s instructions. Proteins were separated by SDS-PAGE and transferred onto polyvinylidene difluoride membranes (Pall Corporation, Port Washington, NY, USA) according to standard protocols. Membranes were immunoblotted with antibodies against HRAS (Merck Millipore, Billerica, MA, USA), TP53, p21, SV40 T antigen, and β-Actin (Santa Cruz Biotechnology, Dallas, TX, USA) in 3% bovine serum albumin in TBST. After primary antibody incubation, the membranes were washed and probed with horseradish peroxidase-conjugated goat anti-mouse or anti-rabbit IgG secondary antibodies (Pierce Biotechnology, Rockford, IL, USA). β-Actin was used as a loading control.

### Canine TP53 target sequencing

Genomic DNA was extracted from canine fetal fibroblasts by using a Wizard Genomic DNA Extraction Kit (Promega, Madison, WI, USA), according to the manufacturer’s instructions. Next, exon 3 of *TP53* in each cell line was amplified using Takara Ex taq**™** (Takara Bio Inc., Shiga, Japan) and primer sets, forward 5′-CTGGTAAGGACTGGGTGTGG-3′ and reverse 5′-GCCACTGACCGTCCAAGTAA-3′under the following conditions in the thermal cycler: 60 s at 95 °C; 30 cycles of: 30 s at 95 °C, 40 s at 58 °C, and 60 s at 72 °C; followed by 10 min at 72 °C. Each product was ligated into a pGEM T-easy vector (Promega), according to the manufacturer’s instructions, and then sequenced using an ABI BigDye® terminator v3.1 Cycle Sequencing kit (Applied Biosystems, Foster City, CA, USA).

### In vivo tumorigenesis assay

To establish subcutaneous xenograft models, 2 × 10^6^ cells of each cell line in phosphate-buffered saline were mixed with 50% Matrigel (Invitrogen, Carlsbad, CA, USA) and transplanted subcutaneously into 5–6-week-old BALB/c nu/nu mice. The size of each tumor was calculated using the following formula: tumor volume (mm^3^) = longest diameter of tumor (mm) × shortest diameter of tumor (mm)^2^/2.

### Karyotyping

Normal canine and *TP53* KO fibroblasts were karyotyped commercially by GenDix, Inc. (Seoul, Korea). The results of karyotyping of the two fibroblast lines were analyzed using the ChIPS-Karyo program, a chromosome image processing system. Analysis data and all terms were represented by an International System for Human Cytogenomic Nomenclature 2016 (ISCN 2016).

### Surveyor assay

Potential off-target genes of each gRNA were predicted using the BLAST algorithm and final target genes for the surveyor assay were selected in accordance with the matched number of nucleotides to each *TP53* gRNA (≥10 of 20) and presence of a NGG PAM sequence.

Genomic DNA was extracted from canine fetal fibroblasts and *TP53* KO cells using a Wizard Genomic DNA Extraction Kit (Promega) according to the manufacturer’s instructions. The surveyor assay was conducted using the Guide-it**™** Mutation Detection Kit (Clontech Laboratories, Inc.) according to the manufacturer’s instructions. During the amplification step, PCR was performed using 100 ng of extracted canine genomic DNA. Primer sequences are listed in Additional file: Table S2.

### Ovulation determination

Unless otherwise indicated, all reagents were obtained from Sigma-Aldrich. All donors and recipients employed in the study showed spontaneous estrous. The estrous stage was examined weekly by observing for vulval bleeding to detect the onset of the heat period. During heat, a 2 mL blood sample was collected daily by cephalic venipuncture and serum P4 levels in the blood samples were measured by electrochemiluminescence immunoassay (Cobas e411, Roche Diagnostics, Mannheim, Germany; intra- and inter-assay coefficients of variation < 4%). Ovarian ultrasonographies were periodically performed twice per day when serum P4 levels were found to be increased by more than 2 ng/mL. The time of ovulation was designated as the time when the ovaries became difficult to find for an apparent decrease in the number or contour of anechoic follicles, or for their disappearance anechogenicity by transabdominal ultrasonography and as the proportion of cornified cells was greater than or equal to 90% of epithelial cells from vaginal swabs, which were stained following Diff Quik (Sysmex Co., Kobe, Japan) standard protocols [38].

### Oocyte collection

All oocyte donors and surrogates underwent spontaneous estrous, and donors and surrogates were matched based on the synchronization of their estrus. Oocytes were surgically retrieved at 3–4 days post-ovulation. Before surgery, a blood sample was drawn through the cephalic venipuncture, and blood plasma was collected and frozen (− 20 °C) for hormone analyses. Anesthesia was induced with a mixture of xylazine hydrochloride (Rumpun®; Bayer Korea, Ansan, Korea; 1 mg/kg body weight) and ketamine HCl (Ketalar®; Yuhan Corporation; 50 mg/mL, Seoul, Korea; 4 mg/kg body weight) and maintained with isoflurane inhalational. Under aseptic conditions, the reproductive tract was exposed through a midventral incision. Corpora lutea (CL) were counted and oocytes were bilaterally flushed from each oviduct with 10 mL TCM 199 supplemented with HEPES (Invitrogen). Oocytes were collected using a stereomicroscope, transferred into fresh medium, and subjected to nuclear transfer.

### Evaluation of retrieved oocytes

The maturation stage of the retrieved oocytes was determined as previously described [39]. The oocytes were stripped of cumulus cells and pre-stained with 5 mg/mL Bisbenzimide (Hoechst 33342) to visualize the presence of nuclei for enucleation process. Oocytes were graded based on their morphology and nuclear stage as immature (cumulus very closely attached to oocytes, nuclear stage is either germinal vesicle (GV), GV breakdown, or metaphase I), mature (M II oocytes with several layers of cumulus cells and homogeneous cytoplasm), aged (unidentified nuclear status with the cytoplasmic membrane shrink, MII oocytes in less than 70% of the cytoplasm and loosely attached cumulus cells,), abnormal (irregular cytoplasmic contour, protrusion of zona pellucida, nuclear immaturity), or ruptured (oocytes with broken zona and cytoplasmic membrane) under an inverted microscope equipped with epiflurescence (TE2000-E; Nikon Corp., Tokyo, Japan).

### Preparation of donor cells

Donor cells originated form dogs active in police or military service. Dermal tissue samples from 2 male Belgian Malinois breeds, and 1 male German, measuring approximately 1 × 3 cm were collected under light tranquilization (Zoletil 50® Virbac, Carros, France) at 0.1 mg/kg and local anesthesia (Daehan lidocaine HCl 2%, Dai Han Pharm Co., Ltd., Seoul, Korea). Sections of subcutaneous tissues were cut into small pieces (approximately 1 × mm^2^) and cultured in DMEM containing 10% FBS at 37 °C in an atmosphere of 5% CO_2_ and air to obtain fibroblasts. Explants were maintained in the culture until they approached 90% confluence. Cells were then trypsinized and reconstituted at concentrations of approximately 1 × 10^6^ cells per mL, and then cryopreserved in cryovials containing DMEM containing 10% dimethyl sulfoxide.

### Nuclear transfer

After evaluating of the maturation status, metaphase II oocytes were enucleated by squeezing out the first polar body and metaphase II plate into a small amount of surrounding cytoplasm using a glass pipette. Donor cells, TP53KO#30 fibroblasts (passage 4), were prepared and treated using a conventional system of primary cell culture as described previously [40]. Using a fine pipette, a trypsinized cells with a smooth cell surface was transferred into the perivitelline space of an enucleated oocyte. The couplets were equilibrated with 0.26 M mannitol solution containing 0.5 mM of HEPES, 0.1 mM of CaCl_2_, and MgSO_4_ for 4 min. Next, the couplets were transferred to a chamber with two electrodes and covered with mannitol solution. The couplets were fused with two DC pulses of 1.75–1.85 kV/cm for 15 μs using a BTX Electro-Cell Manipulator 2001 (BTX, Inc., San Diego, CA, USA). After simultaneous fusion and activation, a group of 5–6 embryos were cultured in 25 μL microdrops of mSOF covered with mineral oil for 1 h at 39 °C in a humidified atmosphere (5% O_2_, 5% CO_2_, and 90% N_2_) until embryo transfer.

### Embryo transfer and pregnancy diagnosis

Surrogate dogs with estrus matching that of oocyte donors were anaesthetized as described previously using an oocyte retrieval procedure. The ovary with a greater number of corpus lutuea was approached by ventral laparotomy. The fat layer covering the ovary was gently grasped with forceps and suspended with a suture to exteriorize the fimbriated end of the oviduct. Immediately after fusion and activation, all reconstructed embryos were loaded into a tomcat catheter (3.5 Fr × 5.5″; Sherwood Medical, St. Louis, MO, USA) with at least a medium volume (2-4 μL) and gently transferred into the 2/3 distal position of the oviduct through the infundibulum. Pregnancy was confirmed by transabdominal ultrasound with a real-time ultrasonography at 25–30 days after embryo transfer. Ultrasonography was performed either in the standing or dorsal recumbency position using a portable ultrasound machine with a 3.5 MHz curved transducer (Sonace R7; Samsung Medison, Seoul, Korea). Ultrasonographies were repeated every 7 days on pregnant surrogates until term. The sizes and shapes of the chorionic cavities and presence of an embryonic or fetal heartbeat were examined to identify embryonic or fetal death.

### Statistical analysis

All experiments were replicated more than three times. All data were analyzed by one-way ANOVA (analysis of variance) followed by Duncan’s test using SPSS software (SPSS, Inc., Chicago, IL, USA) and are reported as the mean ± standard error of the mean. Differences were considered significant if the *P-*value was less than 0.05.cancers and even for testing new anti-cancer therapeutics for dogs.

## Additional file

Additional file 1: **Figure S1.** Representative images of isolated canine fibroblasts with an extended cellular life span and morphologies targeted after transfection of CRISPR/Cas9 vectors of each TP53 gRNA#30, #39, and #51. **Figure S2.** Surveyor assay to identify indel mutations in on-target TP53 or off-target in candidate gene loci in TP53KO#30 and TP53KO#39 cells. **Table S1.** Possible off-target genes of #30 and #39 TP53 gRNA. **Table S2.** Primer sets for Surveyor assay. (PDF 280 kb)
