# Supplementary material for: Establishment of TP53-knockout canine cells using optimized CRIPSR/Cas9 vector system for canine cancer research
Source: BMC Biotechnol. 2019 Jan 3;19:1. doi: 10.1186/s12896-018-0491-5 (PMC6318917; doi:10.1186/s12896-018-0491-5)
Supplement: Supplementary file 1 — Figure S1. Representative images of isolated canine fibroblasts with an extended cellular life span and morphologies targeted after transfection of CRISPR/Cas9 vectors of each TP53 gRNA#30, #39, and #51. Figure S2. Surveyor assay to identify indel mutations in on-target TP53 or off-target in candidate gene loci in TP53KO#30 and TP53KO#39 cells. Table S1. Possible off-target genes of #30 and #39 TP53 gRNA. Table S2. Primer sets for Surveyor assay. (PDF 280 kb) [file 12896_2018_491_MOESM1_ESM.pdf]

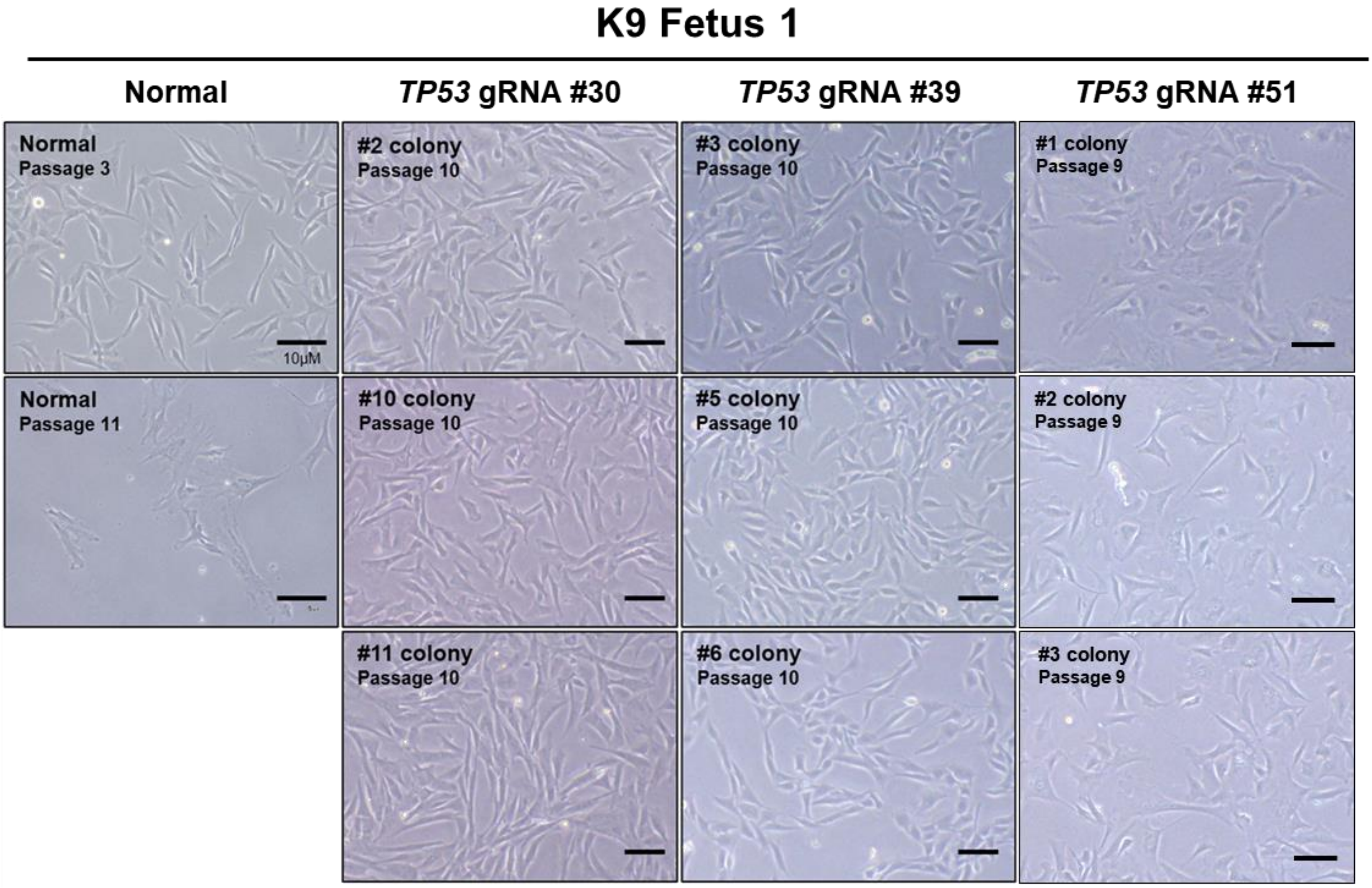

Figure S1. Representative images of isolated canine fibroblasts with an extended cellular life span and normal morphologies targeted after transfection of CRISPR/Cas9 vectors of each *TP53* gRNA #30, #39, and #51.

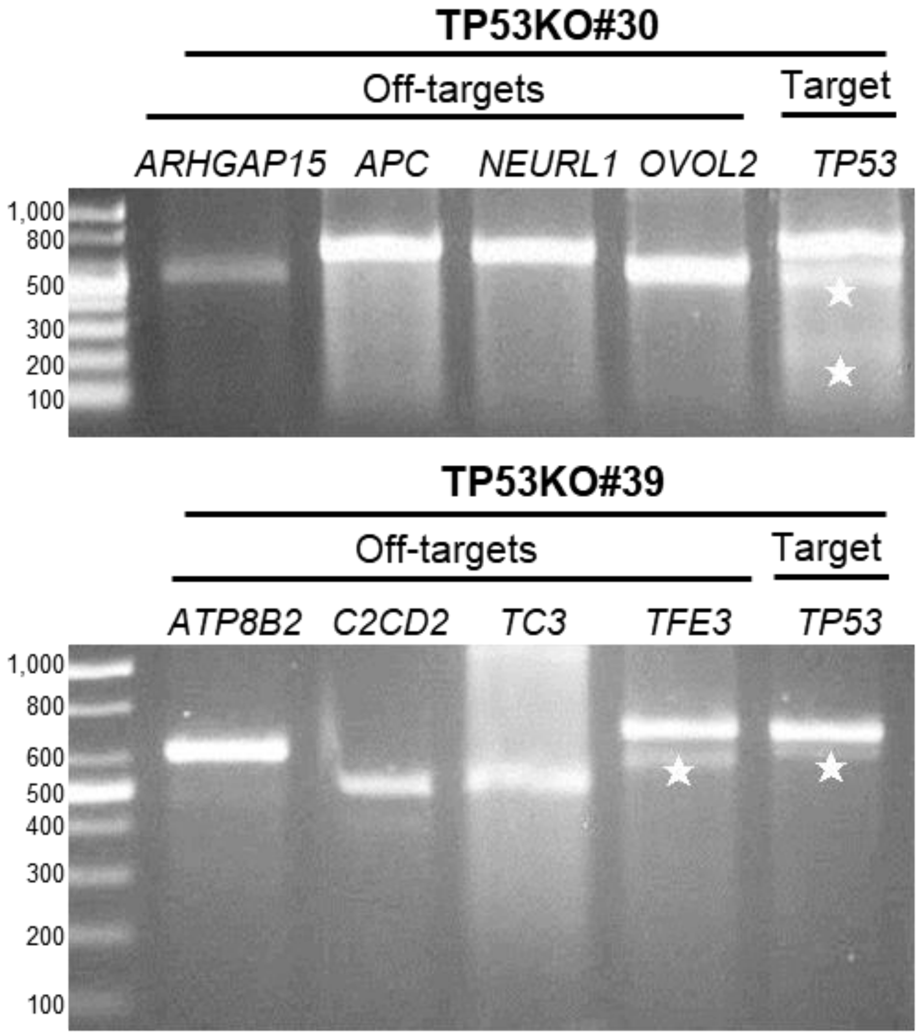

Figure S2. Surveyor assay to identify indel mutations in on-target TP53 or off-target in candidate gene loci in TP53KO#30 and TP53KO#39 cells.

Table S1. Possible off-target genes of #30 and #39 TP53 gRNA

Guide RNA	On & off-target gene				BLAST Score (Identity)
	Symbol		Sequence	PAM	
#30 TP53 gRNA	*TP53*	gRNA	TAGGTGCCAGGGTAGGTCTT	-	46.1 bits (20/20)
		Genome	TAGGTGCCAGGGTAGGTCTT	CGG	
	ARHGAP15	gRNA	CAGGG**T**AGGTCTT	-	24.3 bits (12/20)
		Genome	CAGGG**A**AGGTCTT	CGG	
	APC	gRNA	GGTAGGTCTT	-	26.3 bits (10/20)
		Genome	GGTAGGTCTT	TGG	
	NEURL1	gRNA	GGGTAGGTCTT	-	28.2 bits (11/20)
		Genome	GGGTAGGTCTT	TGG	
	OVOL2	gRNA	GGTAGGTCTT	-	26.3 bits (13/20)
		Genome	GGTAGGTCTT	GGG	
#39 TP53 gRNA	*TP53*	gRNA	TCCCAGAGAGCGTCGTGAAC	-	46.1 bits (20/20)
		Genome	TCCCAGAGAGCGTCGTGAAC	TGG	
	ATP8B2	gRNA	GAGCG**T**CGTGAAC	-	24.3 bits (12/20)
		Genome	GAGCG**A**CGTGAAC	TGG	
	C2CD2	gRNA	AGAGCG**T**CGTGAAC	-	26.3 bits (13/20)
		Genome	AGAGCG**C**CGTGAAC	GGG	
	TC3	gRNA	CCAGA**G**AG**C**GTCGTGAAC	-	26.3 bits (16/20)
		Genome	CCAGA**C**AG**A**GTCGTGAAC	GGG	
	TFE3	gRNA	GAGCGTCG**T**GAAC	-	24.3 bits (12/20)
		Genome	GAGCGTCG**G**GAAC	AGG	

The Bit score is derived from the raw alignment score evaluated by the BLAST search algorithm.
Bolded and underlined capitals show discordant nucleotides b/w gRNA and genomic sequence.

Table S2. Primer sets used for Surveyor assay

Target Gene	Primer Sequence	
TP53	Forward	5′-TGACCCTTGACTCTGGTCTC-3′
	Reverse	5′-TCGGGGTCTATTCCAGAAGC-3′
ATP8B2	Forward	5′-AATCACTCTCACCACGGTCG-3′
	Reverse	5′-CACCGTCACTCCTTGAGAGG-3′
TFE3	Forward	5′-CTGAGATTCAGCCTGGTTCC-3’
	Reverse	5′-GAACACCTTGCAACCCCTAA-3′
TC3	Forward	5′-GTACGGCTTCACTCCCGAT-3′
	Reverse	5′-CCGTCCTGTAAGACAGGCAT-3′
C2CD2	Forward	5′-TGCAGGAGGCATTTTAAGGA-3′
	Reverse	5′-TCACGGGATGCTCTTTAGTT-3′
ARHGAP15	Forward	5′-TGTGGCAACAGAGCCAATA-3′
	Reverse	5′-TTTCAATGACACTGCGTTGC-3′
OVOL2	Forward	5′-AGTAGCAGCCCATCTTCACT-3′
	Reverse	5′-CATCCGAAGAGTTGGAGCTT-3′
NEURL1	Forward	5’-AGGGACAGCCTCAGTGTAG-3′
	Reverse	5′-AGCTAGACTCCCCTCCTTCA-3′
APC	Forward	5’-ACCTATACCACAGAGCGCC-3′
	Reverse	5′-ATGCCTGAGGTTTACTGGGT-3′
